# Mendelian randomization reveals interactions of the blood proteome and immunome in mitral valve prolapse

**DOI:** 10.1038/s43856-024-00530-x

**Published:** 2024-06-06

**Authors:** Louis-Hippolyte Minvielle Moncla, Mewen Briend, Mame Sokhna Sylla, Samuel Mathieu, Anne Rufiange, Yohan Bossé, Patrick Mathieu

**Affiliations:** 1 Genomic Medicine Laboratory, Quebec Heart and Lung Institute, Laval University, Quebec City, QC Canada; 2 Department of Molecular Medicine, Laval University, Quebec City, QC Canada; 3 Department of Surgery, Laval University, Quebec City, QC Canada

**Keywords:** Heritable quantitative trait, Cardiovascular diseases

## Abstract

**Background:**

Mitral valve prolapse (MVP) is a common heart disorder characterized by an excessive production of proteoglycans and extracellular matrix in mitral valve leaflets. Large-scale genome-wide association study (GWAS) underlined that MVP is heritable. The molecular underpinnings of the disease remain largely unknown.

**Methods:**

We interrogated cross-modality data totaling more than 500,000 subjects including GWAS, 4809 molecules of the blood proteome, and genome-wide expression of mitral valves to identify candidate drivers of MVP. Data were investigated through Mendelian randomization, network analysis, ligand-receptor inference and digital cell quantification.

**Results:**

In this study, Mendelian randomization identify that 33 blood proteins, enriched in networks for immunity, are associated with the risk of MVP. MVP- associated blood proteins are enriched in ligands for which their cognate receptors are differentially expressed in mitral valve leaflets during MVP and enriched in cardiac endothelial cells and macrophages. MVP-associated blood proteins are involved in the renewal-polarization of macrophages and regulation of adaptive immune response. Cytokine activity profiling and digital cell quantification show in MVP a shift toward cytokine signature promoting M2 macrophage polarization. Assessment of druggability identify CSF1R, CX3CR1, CCR6, IL33, MMP8, ENPEP and angiotensin receptors as actionable targets in MVP.

**Conclusions:**

Hence, integrative analysis identifies networks of candidate molecules and cells involved in immune control and remodeling of the extracellular matrix, which drive the risk of MVP.

## Plain language summary

One cause of heart disease is mitral valve prolapse, where heart valve thickening leads to malfunction. Biological factors that contribute to this occurrence are largely unknown. We took advantage of different public resources and independent datasets to conduct different converging analyses to identify relevant biological factors. Using genetic variation, we implemented a technique to assess the role of circulating blood proteins on the risk of the disease. We report that blood proteins involved in the regulation of the immune response promote a dysfunctional tissue repair process of the mitral valve. This study has highlighted a contribution of blood proteins that promote excessive tissue repair leading to mitral valve dysfunction. Several of the identified proteins are potential pharmacological targets that could be singled out in future efforts to halt the progression of the disease.

## Abstract (web summary)

Moncla et al. scrutinize the role of circulating proteome in mitral valve prolapse development. Integration conducted on large-scale cross-modality data uncover a causal role of the circulating proteome, immunome and macrophage M2 polarization in mitral valve prolapse.

## Introduction

Mitral valve prolapse (MVP) is a frequent heart valve disorder associated with mitral regurgitation, heart failure and sudden death^1^. MVP is characterized by a thickening of the mitral valve leaflets (MVLs), excess of extracellular matrix (ECM) proteins and glycosaminoglycans (GAGs). These features are often collectively referred to as myxomatous valve degeneration^2^. The underpinning molecular processes are still largely unknown. Rare familial and syndromic disorders have been associated with MVP^3^. Mutations of *FLNA*^4^, *DCHS1*^5,6^, *DZIP1*^7^, and *TNS1*^8^ have been associated with familial and sporadic cases of MVP. MVP is also a manifestation of different syndromes such as Marfan disease, which is caused by a mutation in *FBN1*^9,10^. Recently, large-scale genome-wide association study (GWAS) including 4884 cases of MVP and 434,649 controls has identified 14 risk loci^11^. Though the causal genes are still largely unknown at many risk loci, the latter study identified genomic regions associated with transforming growth factor beta (TGF-β) signaling (*LTB2*, *TGFB2*)^11^, a pathway involved in the remodeling of the ECM. These data are in line with previous works performed in explanted valves, which underlined activation of the TGF-β pathway in myxomatous mitral valves^12,13^. Various alterations of the immune system have been associated with MVP. In explanted human valves, histopathological analysis has underscored that MVLs from MVP were associated with increased levels of macrophages and T cells^14,15^. Also, the presence of autoantibodies has been described in some individuals with MVP^16–18^. In rodents, knock-in of the Flna-P637Q mutation recapitulates some important features of MVP and analysis of the mitral valve transcriptome showed enrichments in chemotaxis and immune cell migration^19^. In dogs, some breeds have a high incidence of MVP and studies showed various associations with the immune response in afflicted animals^20,21^. However, the association between the immune response and MVP is based on observational and fragmentary data.

Rapidly evolving high-throughput technology provides a unique opportunity to integrate cross-modality data in order to capture underlying molecular processes involved in the development of disorders^22–24^. The assessment of gene expression at the genome-wide level and the integration of these data with various analytical pipelines has contributed to underline gene signatures and regulatory pathways associated with traits and disorders^25,26^. Genotyping along with high-throughput assessment of phenomes has underscored the genetic contribution for thousands of molecular and cellular traits^27^. As such, large-scale datasets including expression quantitative trait loci for the blood proteins have been generated^28,29^. These data constitute an important resource to examine causal relationships by using a Mendelian randomization (MR) framework^30,31^. In MR, alleles associated with the exposition (e.g., molecular or cell phenome) and outcome (e.g., traits or disorders) are used as instrumental variables in order to make causal inference^32^. Contrary to observational studies, as alleles are randomly allocated at the time of conception and long before the manifestation of the outcome, MR is not prone to reverse causality bias^33^. Hence, MR is a powerful method to infer causality and identify culprit molecular and cellular phenomes associated with the risk of disorders. Integration of MR data at systems-level into networks and whole-genome gene expression obtained from control and diseased tissues provides a solid framework to explore, with a holistic approach, the pathways involved in different traits and disorders^34,35^. Herein, we leveraged large-scale GWAS, blood proteome-wide data and genome-wide gene expression to perform MR, digital cell quantification, cytokine activity profiling and cross-data integration into networks as well as prediction of ligand-receptor interactions to capture the blood phenome associated with MVP. The present data revealed an unsuspected contribution of the circulating proteome and immunome involved in complex interactions with cells of the mitral valve which drive the risk of MVP. Among the different findings, we underscored that several circulating candidate molecules were predicted to drive the renewal-polarization of macrophages and control the adaptative immune response. We identified culprit actionable targets to modulate the immune response and the remodeling of the mitral valve in MVP.

## Methods

### Genome-wide association study for MVP

Summary statistics for a GWAS including 4884 cases of MVP and 434,649 controls were downloaded from the Cardiovascular Disease Knowledge Portal (URL in data availability section)^11^. Participants were recruited from six cohorts-biobanks. MVP cases were identified according to imaging data and when unavailable the ICD code or by using natural language processing. According to imaging in echocardiographic analysis, a displacement of MVL ≥ 2 mm beyond the mitral annulus in long-axis at end-systole was considered as MVP case. Genetic data were analyzed with age, sex, genotyping array, and principal components as covariates. The study reported 8,269,717 gene variants. Genomic coordinates in GRCh38 were converted in GRCh37 by using LiftOver tool from UCSC.

### Proteome-wide Mendelian randomization

Summary statistics from deCODE genetics encompassing genetic association data performed for 4719 blood proteins measured by an aptamer-based method (SomaScan) (4907 aptamers) in 35,559 Icelanders were downloaded^29^ (build hg38). Also, summary statistics from SCALLOP, which measured 90 cardiovascular blood proteins by an antibody-based method (Olink) in 30,931 subjects were downloaded^28^ (build hg19). Data from deCODE and SCALLOP did not present any cohort overlap with the outcome GWAS or between them. Two-sample MR was performed by using at least three *cis*-independent instrumental variables (*P* < 1 × 10^−05^ and window of 250 kb from the transcription start site). Independent instrumental variables (IVs) (*r*^2^ < 0.1) were identified with PLINK1.9 based on genotypes from European populations from the 1000 Genome project. Rare variants presenting a minor allele frequency inferior to 1% were excluded (MAF < 0.01). For replication analysis, variants presenting a minor allele frequency inferior to 5% were excluded (MAF < 0.05). We performed inverse variance weighted MR and as sensitivity analyses we used the weighted median MR, which allows the use of up to 50% of invalid instruments^36^. The F-statistic was calculated for each instrument using formula *β*^*2*^*/SE*^*2*^^37^^,^. Significance of multiple test correction was established at FDR < 0.05. FDR was calculated by using the R package multtest with the Benjamini and Hochberg test. Bonferroni multiple correction test was conducted at 95% confidence using p.adjust function with Bonferroni method from package Stat in R. Heterogeneity was evaluated by using the Cochran’s Q test. Pleiotropy was evaluated using Egger intercept test. MR analyses were performed by using the Mendelian Randomization package.

To reduce the risk of reverse causation we applied Steiger filtering and Steiger MR^38^. Analyses were performed using the TwoSampleMR package.

### Enrichments analyses

Enrichment analyses were performed by using data from InnateDB^39^, the cardiac gene expression dataset from the Human Protein Atlas^40^ and ligand-receptor pairs as reported by ref. ^41^. Data in the Human Protein Atlas from the *Tissue cell type section* and the *Heart muscle* in the *enriched* category were downloaded for processing in enrichment analyses (URL is provided in Data availability section). Manual curation for ligands-receptor pairs was also performed to ensure that novel or non-reported interactions with the blood proteome were retrieved (IL16 with CD9)^42,43^. Hypergeometric tests were performed by using the python library scipy.stats and using a curated gene list formed from the unique intersection of deCODE and SCALLOP as the background: 4316 unique proteins on the autosomes (MVP GWAS reported the autosomes data). Results were visualized using packages ggplot2, tidyverse, circlize and migest in R.

### Pathway and gene ontology

Gene ontology (GO) and pathway (Panther molecular function) analyses were performed with Enrichr^44^ (https://maayanlab.cloud/Enrichr/).

### Network analyses

We used the associated blood proteins as seeds to extract a network from the InnateDB dataset reporting curated protein-protein interactions. Data were visualized using Cytoscape 3.9.1^45^ alongside StringApp^46^ and Enhanced graphics^47^ addons. Metrics and algorithms including degree centrality, cliques and voterank, were determined by using the python library NetworkX. The walktrap algorithm to identify the network modules was implemented in NetworkAnalyst^48^ (https://www.networkanalyst.ca/). For each network module, we reported the most significant GO (GO molecular function or GO biological process).

### Gene expression and digital cell quantification

Microarray gene expression dataset performed on MVLs obtained from GSE109744 were analyzed. Data were background corrected, normalized by the variance stabilizing normalization (vsn) and differential gene expression analysis was performed with the limma package in R in ExpressAnalyst. For genes tagged with more than one probesets the median value was calculated and used in downstream analyses. Digital cell quantification was performed by using GEDIT^49^. GEDIT is a python package that implements a cell deconvolution algorithm to infer cell population proportions from a reference dataset. We implemented GEDIT by using the immune LM22 reference dataset and by using the default settings. Groups were compared with unpaired Student’s t-test in R. Principal component analysis was performed with singular value decomposition and using a unit of variance as the scaling method in R package pcaMethods. Graphs were generated by using gglot2 and tidyverse in R.

### GSEA

Analysis was conducted by using toolkit Webgestalt^50^ (https://www.webgestalt.org/). Gene expression from MVLs microarray dataset (GSE109744) were ordered on their fold change (MVP vs. control). Resulting list was used for gene set enrichment analysis (GSEA). Analysis was conducted by using the Reactome pathway database. Resulting enrichments terms were sorted and filtered at FDR < 5%.

### Cytokine activity profiling

Gene expression pattern in microarray data of control and MVP from GSE109744 were used to conduct a cytokine activity profiling analysis for 43 cytokines and growth factors using Cytokine Signaling (CytoSig)^51^ (https://cytosig.ccr.cancer.gov/). The algorithm quantifies the cytokine activity from the gene expression pattern in a query tissue-cell by using regression analysis against a composite cytokine response profile obtained experimentally. The experimentally-derived cytokine signatures have been obtained by automatic (artificial intelligence) and manual curation of 20,591 transcriptome profiles from RNA-seq and microarray datasets. In the CytoSig framework, predicted cytokine activity is the z-score of the ridge regression coefficient from the query expression pattern on the composite signatures obtained experimentally from 20,591 transcriptome profiles. Pairwise correlation (Pearson) was performed on the cytokine activity profiling obtained in MVLs and represented with hierarchically clustering in a dendrogram; height was indexed on the Euclidian distance. Differential cytokine activities were identified by using Wilcoxon rank-sum test between control and MVP MVLs; statistics were performed in R. Data were represented as ridgeline graph. Response data to CSF1 were retrieved from meta-analysis performed by Cytokine Signaling (CytoSig) and were represented as boxplots. Dendrogram was generated using libraries tidyverse, cluster, factorextra, dendextend and base stats in R. Ridgeline diagram was generated using libraries ggplot2 and ggridges in R. Boxplots were generated using ggplot2.

### Identification of actionable targets

We interrogated DGIdb^52^ (https://www.dgidb.org/), an extensive repository of drug-gene pairs collated through different resources to assess actionable targets from the blood proteins identified in MR. We considered approved and in-development drugs. Additionally, manual curation of the literature was performed for each candidate blood protein in order to extend the assessment of actionable targets. A target was deemed actionable if reported in DGIdb or in the literature and were in-development or approved drugs.

### Ethics approval and consent to participate

Datasets used in this study are comprised of human participants. Datasets were publicly available, and no ethical approval was necessary for secondary use of this data.

### Reporting summary

Further information on research design is available in the Nature Portfolio Reporting Summary linked to this article.

## Results

**Fig. 1:**
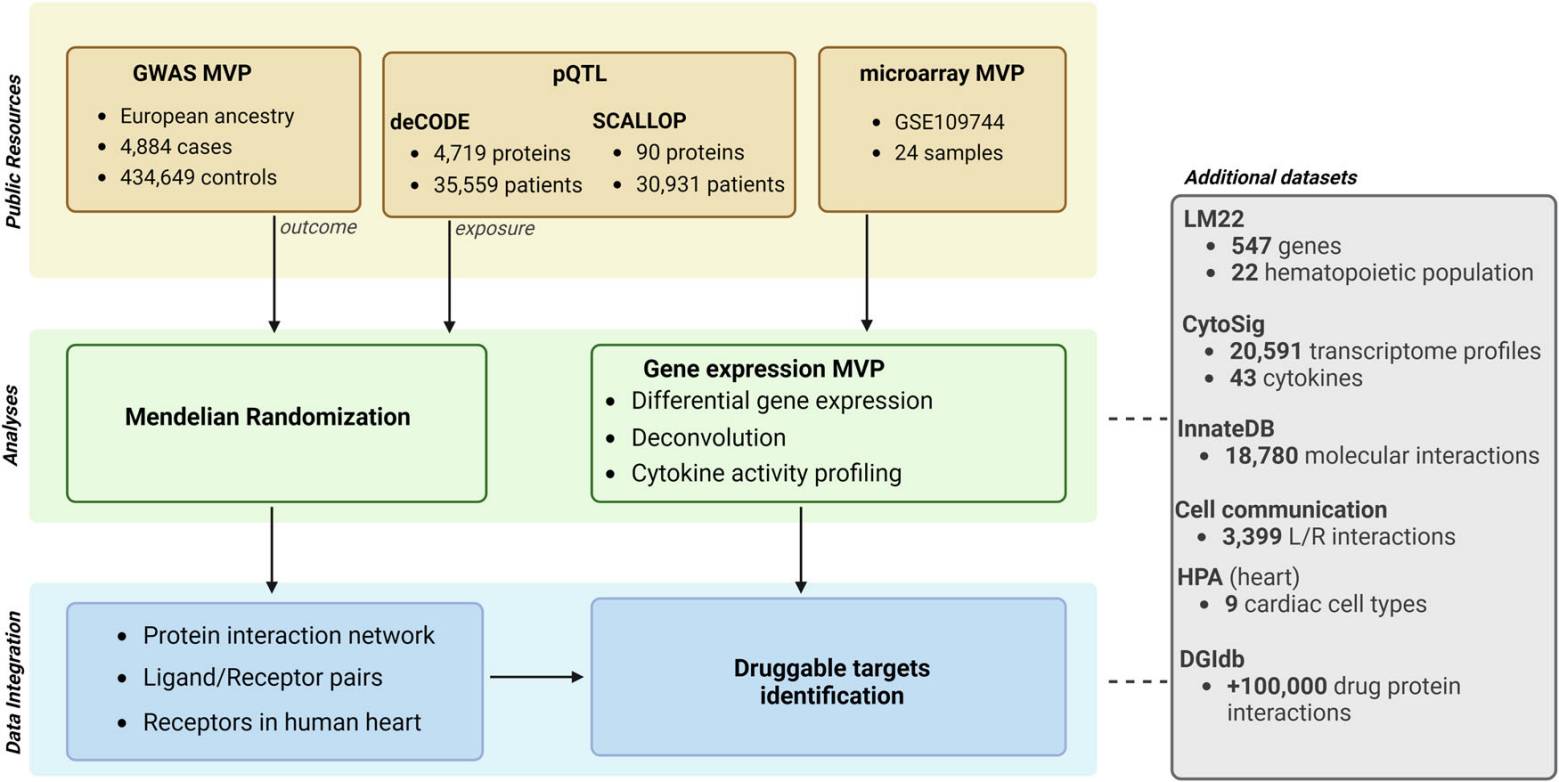
Analytical pipeline. Schematic representation of the analytical pipeline, which is divided in three main parts. The first part represents the public resources used in this study, namely the GWAS MVP, the protein quantitative trait loci (pQTL) datasets deCODE and SCALLOP as well as the microarray dataset (GSE109744). The second part represents the analytical pipeline. GWAS MVP was used alongside with pQTL data to conduct a causal inference analysis by using Mendelian randomization. Microarray data of human explanted control and MVP heart valves (GSE109744) were used to conduct differential gene expression analysis, deconvolution of immune cell populations (digital cell quantification) and cytokine activity profiling with the help of additional datasets LM22 and CytoSig. Finally, data were integrated into network by using InnateDB. Ligand-receptor (L/R) communication was inferred in cardiac cells of the Human Protein Atlas (HPA) and the mitral valve. Results were processed to identify druggable candidate molecules by using DGIdb.

Figure 1 is a schematic representation of the analytical pipeline.

### Circulating proteome associated with MVP

The blood compartment is at the interface of tissues and organs. It transports multiple proteins, which regulate biological processes^53^. We hypothesized that circulating proteins may participate to the risk of MVP. We took advantage of recently published GWAS data for MVP, which included 4,884 cases and 434,649 controls, to perform a proteome-wide two-sample MR analysis on the risk of MVP^11^. For the exposition, we selected two independent cohorts for which GWAS summary statistics were available. In the first cohort from deCODE, 4719 blood proteins were measured by an aptamer-based method in 35,559 individuals^29^. In the second cohort from the SCALLOP consortium, 90 blood proteins were measured by a multiplex immunoassay-based method in 30,931 subjects^28^.

**Fig. 2:**
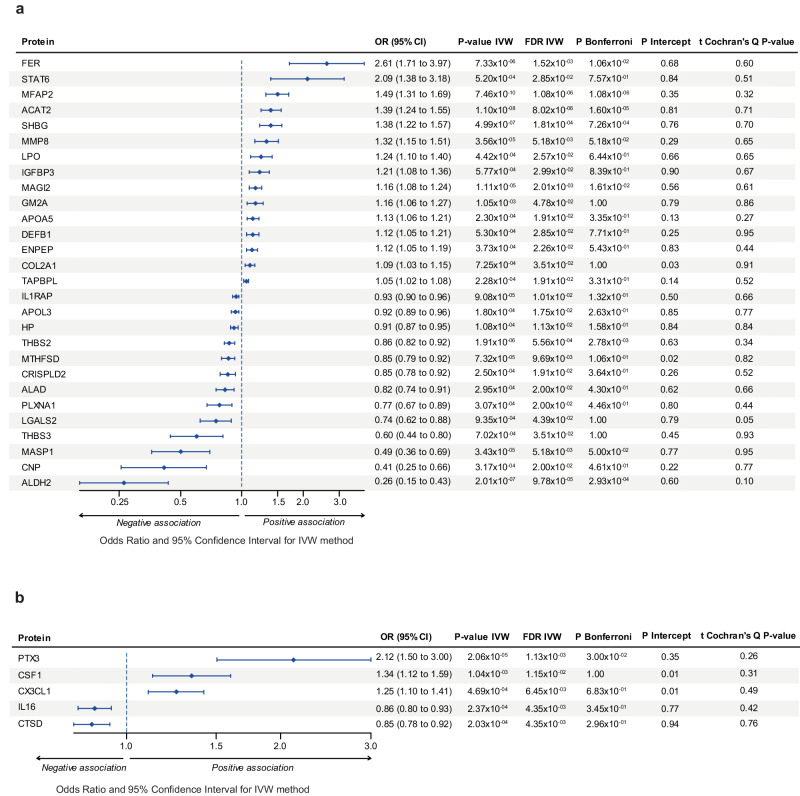
Blood proteins and immune traits significantly associated with MVP. **a** Forest plot illustrating significant MR associations between blood proteins from deCODE and MVP with multiple correction test FDR and Bonferroni as well as heterogeneity and pleiotropy tests. **b** Forest plot illustrating significant MR associations between blood proteins from SCALLOP and MVP with multiple correction test FDR and Bonferroni as well as heterogeneity and pleiotropy tests. OR odd ratio, 95%CI confidence intervals at 95%, IVW inverse variance weighted.

We performed MR analyses independently in each cohort for the exposition by selecting at least three independent *cis*-instrumental variables (*P* < 1 × 10^−05^) with minor allele frequency (MAF) ≥ 1% (methods). In the deCODE cohort, enough instruments (≥3) (median instrumental variables: 17) were identified for 1455 blood proteins and for which inverse variance weighted MR (IVW-MR) was performed. After correction for multiple testing, 32 blood proteins were associated with MVP at false discovery rate (FDR) < 5% (Supplementary Data 1). From these proteins, 31 did not show heterogeneity (*t* Cochran’s Q *P* value > 0.05) and only two showed modest risk of pleiotropy (*P*_Intercept_ <0.05), namely COL2A1 and MTHFSD with *P*_Intercept_ at 0.03 and 0.02, respectively. As a sensitivity measure we implemented weighted median MR (WM-MR), which is resistant to pleiotropy allowing up to 50% of invalid instruments^36^. In WM-MR, 28 unique blood proteins remained associated with MVP (ACAT2, ALAD, ALDH2, APOA5, APOL3, CNP, COL2A1, CRISPLD2, DEFB1, ENPEP, FER, GM2A, HP, IGFBP3, IL1RAP, LGALS2, LPO, MAGI2, MASP1, MFAP2, MMP8, MTHFSD, PLXNA1, SHBG, STAT6, TAPBPL, THBS2, THBS3) (FDR_WM-MR_ < 0.05) (Fig. 2a) (Supplementary Data 2).

By using the SCALLOP cohort as the exposition, there were at least three instrumental variables (median instrumental variables: 11) to perform 55 MR analyses (Supplementary Data 3). After filtering the data for the same criteria as above (FDR_IVW-MR_ < 0.05, t Cochran’s Q *P* value > 0.05, FDR_WM-MR_ < 0.05), five blood proteins (CSF1, CTSD, CX3CL1, IL16, PTX3) remained associated with the risk of MVP **(**Fig. 2b) and two of which presented mild risk of pleiotropy (*P*_Intercept_ = 0.01 for CX3CL1 and CSF1) and thus remained selected as plausible causal candidate proteins (Supplementary Data 4).

For each dataset, the F-statistic for the instruments was ≥20, which is well above the suggested threshold of 10 to perform MR^37^. Hence, by combining the two datasets we identified a total of 33 unique blood proteins robustly (FDR_IVW-MR_ < 0.05, t Cochran’s Q *P* value > 0.05, FDR_WM-MR_ < 0.05) associated with the risk of MVP. Among these blood candidates, 4 proteins identified by SCALLOP (CTSD, CX3CL1, IL16, PTX3) were also part of the deCODE dataset and had enough instruments to carry out MR. From the SCALLOP-identified proteins, CTSD, CX3CL1 and IL16 were also nominally significant in the deCODE dataset (*P*_IVW-MR_ < 0.05) and had consistent directional effects **(**Supplementary Data 5**)**. At a more stringent Bonferroni threshold, eight proteins (MFAP2, ACAT2, ALDH2, SHBG, THBS2, FER, MAGI2, PTX3) were associated with MVP. The strongest association (lowest *P*_IVW-MR_ value) was for MFAP2 (microfibril associated protein 2) (per 1 SD) (OR:1.49, 95%CI:1.31–1.69, *P* = 7.46 × 10^−10^).

Additional sensitivity MR analyses were conducted with more conservative MAF threshold. By using IVs with MAF ≥ 5%, IVW-MR showed that 32 blood proteins (MFAP2, ALDH2, SHBG, LGALS2, ACAT2, FER, THBS2, CRISPLD2, MTHFSD, TAPBPL, CNP, STAT6, MASP1, MAGI2, DEFB1, LPO, APOL3, IL1RAP, APOA5, MMP8, COL2A1, GM2A, HP, THBS3, ALAD, ENPEP, PLXNA1, CTSD, CSF1, IL16, PTX3, CX3CL1) replicated at FDR_IVW-MR_ < 5% (97% replicated). Only IGFBP3 could not be replicated (Supplementary Data 6 and 7).

To ensure unbiased results, MR-Steiger and filtering method were applied to IVs for the 33 blood protein candidates identified previously. Steiger filtering aim to remove variants that explain more variance for the outcome than the exposure to exclude the possibility of reverse causation. Steiger approach showed that all IVs for the 33 blood protein candidates showed no evidence of reverse causation (Supplementary Table 1 and 2). Hence, the 33 blood proteins (Fig. 2a, b) were considered for downstream analyses.

### Analysis of MVP-associated proteome in evidence-based molecular network

**Fig. 3:**
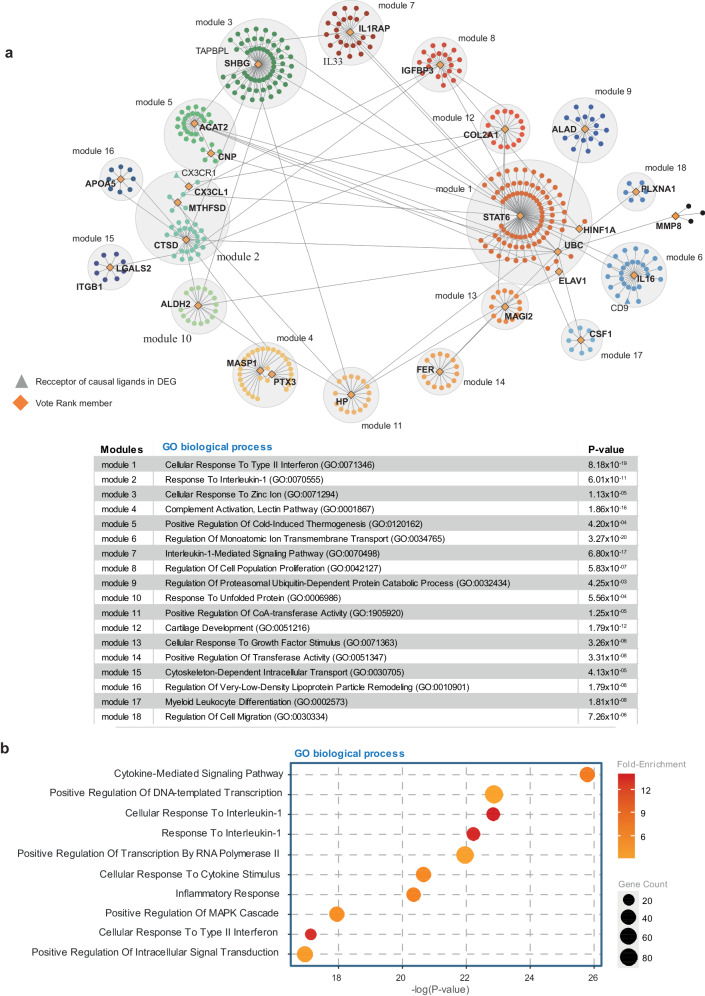
Protein-protein interaction network and modules analysis in MVP. **a** Associated blood proteins were used as seeds to extract a protein-protein network from InnateDB^39^. Modules were determined using the walktrap algorithm and were colored for identification; for each module the most significant GO biological process is indicated; voterank nodes (diamonds) and receptor of associated blood ligands (triangles) are identified in the network. **b** GO biological process enrichment analysis for all nodes in the network.

Previous studies have highlighted the potential role of the immune system in the development of MVP^14,19,20^. However, whether circulating proteins may modulate the immune activity in MVP is presently unknown. We thus examined the overlap of MVP-associated blood proteins with the immune dataset collected by InnateDB. We identified a significant enrichment for MVP-associated blood proteins in the immune response (fold-enrichment:2.18, *P* = 0.02, hypergeometric test) (MASP1, STAT6, LPO, IL16, CX3CL1, CSF1, DEFB1, IL1RAP). To capture the general function of the blood proteins associated with MVP we implemented a network approach. We extracted a network from the InnateDB dataset of protein-protein interactions (PPI), which includes more than 18,000 PPIs, by using MVP-associated blood proteins as seeds. We generated a protein interaction network composed of 440 nodes (proteins) and 469 edges (connections) (Fig. 3a). The network was enriched for gene ontology (GO) in cytokine mediated signaling pathway (GO:0019221) (*P* = 1.64 × 10^−26^, hypergeometric test) (Fig. 3b). In this network, the top 10 most connected proteins (highest degree) were blood MVP-associated proteins (STAT6, SHBG, CTSD, ACAT2, IL16, IGFBP3, IL1RAP, MASP1, HP, COL2A1). As networks are highly modular, with each module encapsulating a function, we implemented an algorithm to identify modules in the MVP network^48^ (methods). The MVP network included 18 different modules, several of which are related to immunity and inflammation in gene ontology **(**Fig. 3a). Some of these modules are enriched in cell or molecular functions. For instance, module 3, which includes SHBG (OR:1.38, 95%CI:1.22–1.57, *P*_IVW-MR_ = 4.99 × 10^−07^), a candidate circulating protein, is enriched for cellular response to zinc ion (GO:0071294) (*P* = 1.13 × 10^−05^). SHBG (sex hormone binding globulin), a blood transporter for androgen and estrogen, is coordinated with a zinc binding site that modulates the affinity for estrogen^54^. In networks, the communication between the different modules is determined by influential nodes (proteins). Biologically, influential proteins propagate information and could thus link different functional modules. We implemented voterank, an iterative algorithm, to identify the most influential proteins from the MVP blood network^55^. From the 440 network nodes, voterank identified 26 blood proteins as influential nodes (CX3CL1, STAT6, CTSD, PTX3, MTHFSD, CSF1, HP, APOA5, COL2A1, CNP, HNF1A, MMP8, IL16, IL1RAP, ELAVL1, ALAD, ALDH2, PLXNA1, SHBG, IGFBP3, LGALS2, FER, ACAT2, UBC, MASP1, MAGI2). These influential blood proteins were enriched in cytokine-cytokine receptor interactions, tryptophan metabolism and fatty acid degradation (Supplementary Table 3). Thus, these data suggested that the circulating proteome may impact several functions, such as cytokine communication, which may impact the mitral valve.

### Characterization of ligand–receptor interactions in the MVP-associated blood proteome, cardiac cells and mitral valve leaflets

**Fig. 4:**
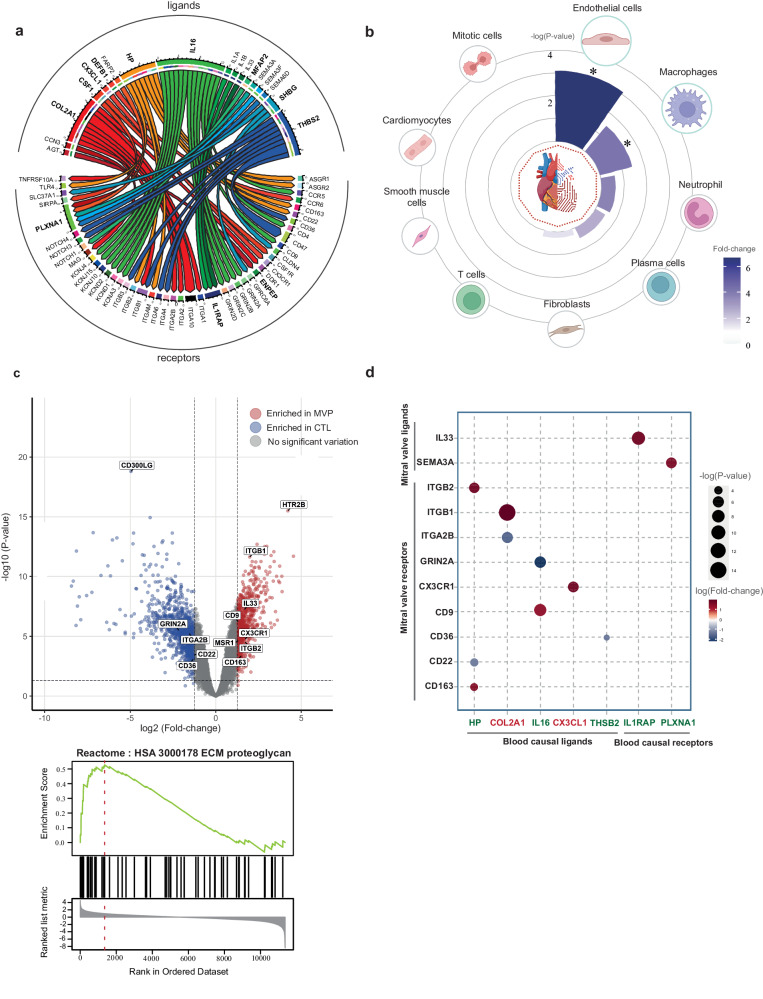
Analysis of ligand–receptor pairs identified from candidate blood proteins. **a** Chord diagram summarizing ligand–receptor pairs identified in candidate causal blood proteins using a comprehensive list of ligand-receptor interactions compiled by ref. ^41^. Bold are candidate blood proteins. **b** Circular plot illustrating the enrichment of receptors for their respective cognate candidate blood ligands in nine different cardiac cell-types from the heart dataset in the Human Protein Atlas^40^. Cell-types are ordered clockwise according to their enrichment *P* values (hypergeometric test); −log (*P* value) is represented on concentric circles and fold-change as color scale; *indicates *P* < 0.05. Figure generated with the help of Biorender. **c** Volcano plot depicting differentially expressed genes between control and MVP MVLs in microarray dataset (GSE109744). Genes were considered differentially expressed at a log2fold-change >1.25 and FDR < 5%. Lower panel represents gene set enrichment analysis (GSEA) in Reactome. **d** Plot depicting inferred ligand-receptor interactions between blood proteins associated with MVP (x-axis; labels in red and green are for positively and negatively associated blood proteins with the risk of MVP respectively) and differentially expressed genes in MVLs (y-axis); fold change (MVP vs. control) is represented on a color scale, whereas the −log (*P* value) is represented as the size of the dots.

The circulating proteome includes a collection of ligands and receptors, which modulate the response of cells^56^. Ligands may activate or inhibit receptors located in various tissues, whereas soluble receptors in circulation may bind to their cognate ligands as decoy factors in limiting cell signaling^57,58^. To assess the role of the circulating proteins identified in MR, we leveraged a comprehensive list of ligand-receptor pairs curated by ref. ^41^. The MVP-associated blood proteome was significantly enriched in ligands (fold-enrichment: 2.04, *P* = 0.02, hypergeometric test). In total, we identified 53 different predicted ligand-receptor pairs involving blood proteins associated with the risk of MVP (Fig. 4a). We next asked whether the ligands of MVP-associated proteome could interact with receptors expressed in cell populations of the heart. We leveraged the heart dataset from the Human Protein Atlas^40^, which provides list of genes enriched in nine different cardiac-specific cell-types^59^ (endothelial cells, fibroblasts, macrophages, mitotic cells, neutrophil, plasma cells, smooth muscle cells, cardiomyocytes, T cells). We found that the receptors to the cognate causal candidate blood ligands were enriched in cardiac endothelial cells (*NOTCH4*, *NOTCH1*, *ITGA1*, *TNFRSF10A*, *TLR4*) (fold-enrichment:6.71, *P* = 7.92 × 10^−04^, hypergeometric test) and cardiac macrophages (*CD163*, *ITGB2*, *ITGAM*) (fold-enrichment:4.21, *P* = 0.03, hypergeometric test) (Fig. 4b). These observations are consistent with our previous results and suggest that MVP-associated blood proteins may interact with cardiac immune cells, namely macrophages.

### Differential expression of ligand-receptor pairs in mitral valve leaflets and interactions with MVP-associated blood proteome

To further assess the potential interactions between the blood proteome and the mitral valve, we analyzed a microarray dataset (GSE109744) comparing genome-wide gene expression between MVLs from control and MVP individuals^60^. There were 1,853 differentially expressed genes (DEG) between MVP and control MVLs (log2fold-change > 1.25, false discovery rate (FDR) < 5%) **(**Fig. 4c). A Reactome GSEA of the microarray showed that MVLs from MVP were enriched in ECM proteoglycans (R-HSA-3000178) (normalized enrichment score:2.19, *P* < 2.2 × 10^−16^), a hallmark feature of MVP (Fig. 4c). Next, we evaluated if the receptors for the candidate ligands in circulation were differentially expressed in MVLs from control and MVP. We found that 9 receptors to the candidate blood ligands were among the DEG in MVLs (*CX3CR1*, *GRIN2A*, *ITGB2*, *CD163*, *CD22*, *ITGB1*, *CD36*, *ITGA2B*, *CD9*) (Fig. 4d). For instance, CX3CL1 (fractalkine) (OR:1.25, 95%CI:1.10-1.41, *P*_IVW-MR_ = 4.69 × 10^−04^) is predicted to interact with CX3CR1, which is overexpressed in MVLs from MVP. HP (haptoglobin) (OR:0.91, 95%CI:0.87-0.95, *P*_IVW-MR_ = 1.08 × 10^−04^) and IL16 (OR:0.86, 95%CI:0.80-0.93, *P*_IVW-MR_ = 2.37 × 10^−04^), two circulating proteins negatively associated with the risk of MVP, were predicted to interact with ITGB2 (HP), CD163 (HP) and CD9 (IL16), which are overexpressed in MVLs from MVP subjects. Figure 4d illustrates the inferred interactions including the directional effects of circulating causal ligands in MR (on the risk of MVP) and whether their respective receptors were up- or down-regulated in MVLs from MVP (MVP vs. control).

As previously discussed, soluble receptors are involved in the regulation and biological activity of ligands^61,62^. The identification of their cognate ligands in MVP could lead to a better understanding of the pathology. Among the circulating soluble receptors associated with MVP, IL1RAP and PLXNA1 interact with ligands for which expression is dysregulated in MVLs from MVP. Soluble IL1RAP (sIL1RAP) (OR:0.93, 95%CI:0.90-0.96, *P*_IVW-MR_ = 9.08 ×10^−05^), which is negatively associated with the risk of MVP, is a decoy factor for IL1B as well as IL33 and limits the downstream signaling from these cytokines^63^. In MVLs from MVP individuals, the expression of *IL33*, a cytokine promoting M2 polarization, was increased^64^ (log2fold-change:1.67, *P* = 1.77 × 10^−09^) (Fig. 4d). Soluble PLXNA1 (sPLXNA1) (OR:0.77, 95%CI:0.67–0.89, *P*_IVW-MR_ = 3.07 × 10^−04^) is associated with a lower risk of MVP and binds to class 3 semaphorins (SEMA3A-F), which are secreted^65^. In MVLs from MVP, the expression of SEMA3A (log2fold-change:1.56, *P* = 2.04 × 10^−06^) is increased, whereas expression of PLXNA1 (log2fold-change: −1.45, *P* = 7.10 × 10^−07^) is decreased. Though the role of sPLXNA1 has not yet been investigated it is possible that it modulates biological activity of SEMA3A, which is involved in immune regulation^66^. Hence, these data suggest that communications between genetically predicted blood protein levels and dysregulated gene expression in MVLs may participate to MVP.

### Immune cell quantification and cytokine activity profiling in MVP

**Fig. 5:**
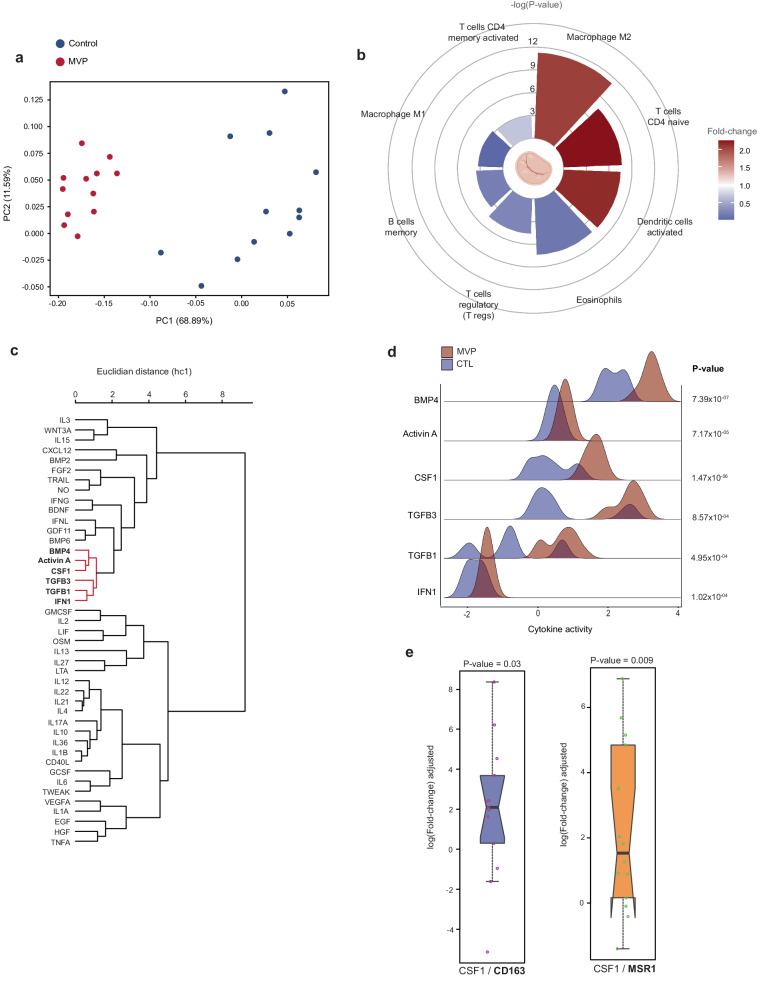
Digital cell quantification and cytokine activity profiling. **a** Principal component analysis (PCA) of digital cell quantification for immune cells in MVLs. **b** Circular plot representing significant enrichment for the immune cell type proportions (MVP vs. control MVLs); −log (*P* value) is represented on concentric circles and fold-change as color scale; data illustrated are FDR < 0.05. Figure generated with the help of Biorender. **c** Dendrogram showing similarity profile for cytokine activities in MVLs. In red are similar cytokine activity profiles with involvement in the polarization of macrophage. **d** Ridgeline plot comparing cytokine signature activities in control (CTL) and MVP leaflets for BMP4, Activin A, CSF1, TGFB3, TGFB1, and IFN1 with corresponding *P* values. (*n* = 12 MVP samples; *n* = 12 control samples) **e** Boxplot representing a meta-analysis of differential expression of gene targets *CD163* and *MSR1* in response to treatment with CSF1. Each dot represents a treatment response for an experiment performed in human blood monocytes or hematopoietic stem cells available in the CytoSig database (CD163 *n* = 13 independent experiments; MSR1 *n* = 14 independent experiments).

Considering that immune cells are present in MVLs from MVP^14,15^ we wondered whether the proportion of immune cells could be altered in diseased heart valves and play a role in the development of MVP. To validate this hypothesis, we performed digital cell quantification (DCQ) using microarray data on MVP and control MVLs (GSE109744). DCQ uses a computational algorithm to estimate the proportion of different cell types from bulk genome-wide gene expression^67^ using reference datasets of specific cell types. We implemented DCQ by using a validated compendium of cell-specific gene expression profile for 22 human immune cell types^68^ (LM22) and a deconvolution algorithm to infer the proportion of cells in MVLs^49^. In principal component analysis, the proportion of cells obtained from DCQ provided a good discrimination between control and diseased MVP leaflets (Fig. 5a). Figure 5b represents the enrichments for the immune cell types in MVLs (MVP vs. control). In MVLs from MVP, DCQ showed a significant (FDR < 5%) shift toward higher proportions of M2 polarized macrophages (0.19 vs. 0.09, *P* = 5.46 × 10^−12^), activated dendritic cells (0.12 vs. 0.05, *P* = 3.13 ×10^−08^) and naive CD4 T cells (0.13 vs 0.06, *P* = 2.16 × 10^−08^) (Fig. 5b). Regarding macrophages, their augmented proportion in MVP and their significant enrichment in cognate receptors to potential causal blood proteins point toward the important role for this cell type in the development of the pathology.

Classically (M1) and alternatively (M2) activated macrophages are dynamically polarized from M0 macrophages. CSF1 (also known as M-CSF), a protein significantly associated with MVP in MR analysis, is a determinant factor promoting the maintenance and differentiation of M0 into M2 macrophages with a potential to differentiate in different subsets including M2a, M2b, and M2c, which are promoted by different sets of cytokines namely IL4/IL13, IL1B, and TGF-β respectively^69^. Taking these results into account, a better understanding of cytokine activity in MVLs may provide further evidence supporting the role of M2 macrophage polarization in MVP. From the gene expression pattern in control and MVP leaflets, we assessed the activity of 43 different cytokines and growth factors according to an algorithm (CytoSig) implementing artificial intelligence assisted by expert curation, which provides an atlas of robust cytokine activity signatures obtained from differentially expressed genes and derived from 20,591 microarray and RNA-seq experiments^51^. The algorithm quantifies the cytokine activity from the gene expression pattern in a query tissue-cell by using regression analysis against a composite cytokine response profile obtained experimentally^51^. Figure 5c shows a similarity profile for the activity of cytokines derived from the gene expression pattern in MVP and healthy MVLs. In line with the MR analysis, we found that the signature activity of CSF1, a blood protein positively associated with the risk of MVP, was significantly increased in MVP compared to control MVLs (Fig. 5d). The activity of CSF1 was correlated with the activities of activin A (INHBA), TGF-beta members, BMP4 and type I interferons, which were increased in MVP (Fig. 5c, d). These cytokines have been shown to promote M2 polarization of macrophages with tissue remodeling properties (M2c subtype)^70–75^. To confirm the role of CSF1 in MVLs, we next interrogated the CytoSig database of transcriptome profiles in response to various cytokine treatments^51^. In response to CSF1, data-driven observations from different experiments performed in human blood monocytes and hematopoietic stem cells showed significant elevations of *CD163* and *MSR1*, two markers of M2c macrophage^76–78^ (Fig. 5e). Accordingly, in MVP the expression of *CD163* (log2fold-change:1.49, *P* = 1.18 × 10^−04^) and *MSR1* (log2fold-change:1.31, *P* = 3.48 × 10^−06^) was significantly increased (Fig. 4c). Collectively, DCQ and cytokine activity profiling provided solid evidence for a role of the MVP-associated blood protein CSF1 (M-CSF) (OR:1.34, 95%CI:1.12–1.59, *P*_IVW-MR_ = 1.04 × 10^−03^) and co-regulated cytokines of the TGF-beta family and type I interferons, which promote the M2 polarization of macrophages with tissue remodeling and ECM synthesis properties.

### Identification of actionable targets in MVP

**Fig. 6:**
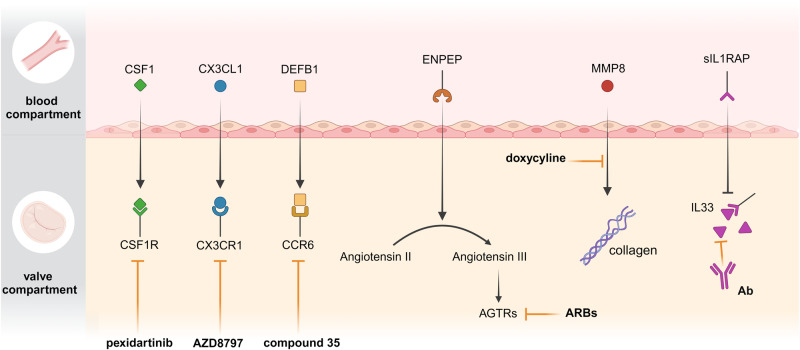
Actionable targets in MVP. Graphical representation of actionable targets when considering the directional effect and the mode of action (ligand vs. soluble receptor) of associated blood proteins (blood compartment) and targets in MVLs (valve compartment). Identified actionable targets are: circulating CSF1 and MVL receptor CSF1R with pexidartinib^79^; circulating CX3CL1 and overexpressed receptor in MVLs CX3CR1 with blocker AZ8797^80^ (currently in development); circulating DEFB1 and MVL receptor CCR6 with inhibitor “Compound 35”^81^ (currently in development); circulating ENPEP with aminopeptidase inhibitor RB150 (in development) and drug repositioning for angiotensin receptor blockers (ARBs)^86,87^; AGTRs: angiotensin receptors; MMP8 with metalloproteinase inhibitor doxycycline; IL33, an interleukin overexpressed in MVP, with its soluble decoy receptor IL1RAP (sIL1RAP) and neutralizing antibodies for IL33^57^. Figure generated with the help of Biorender.

We next evaluated whether the circulating proteins associated with the risk of MVP were potentially actionable for therapeutic developments. We interrogated the Drug Gene Interaction Database (DGIdb), which provides a comprehensive list of drug-gene pairs collated through an extensive list of resources^52^. From DGIdb, 60 drug-gene pairs targeting the MVP-associated blood proteins were identified. According to the directional effects (positively associated with the risk of MVP), 50 drug-gene pairs were deemed potentially actionable (Supplementary Table 4). Figure 6 illustrates the most relevant interactions between the blood proteome and the proteins of MVLs along with their corresponding drugs. Among the immunomodulatory molecules, pexidartinib is a blocker of CSF1R, the receptor for CSF1^79^ (OR:1.34, 95%CI:1.12–1.59, *P*_IVW-MR_ = 1.04 × 10^−03^), and could be repositioned for MVP. Following manual curation, we identified that among the molecules in development, AZD8797, a blocker for CX3CR1^80^, which is overexpressed in MVP leaflets, could represent a suitable target. To this effect, CX3CR1 is the receptor for the candidate circulating agonist CX3CL1 (OR:1.25, 95%CI:1.10–1.41, *P*_IVW-MR_ = 4.69 × 10^−04^). Also, novel under development CCR6 inhibitors^81^ are of interest as we identified that circulating DEFB1 (defensin beta 1) (OR:1.12, 95%CI:1.05–1.21, *P*_IVW-MR_ = 5.30 ×10^−04^), a CCR6 agonist^82^, was associated with MVP. Considering that sIL1RAP (OR:0.93, 95%CI:0.90–0.96, *P*_IVW-MR_ = 9.08 × 10^−05^) is a decoy receptor for the MVP overexpressed gene *IL33*, neutralizing antibodies for IL33 may represent a target to investigate^83^. Although lacking specificity, doxycycline is a metalloproteinase inhibitor^84^, which targets MMP8 (OR:1.32, 95%CI:1.15–1.51, *P*_IVW-MR_ = 3.56 × 10^−05^), a circulating causal candidate in MVP. ENPEP (glutamyl aminopeptidase) (OR:1.12, 95%CI:1.05–1.19, *P*_IVW-MR_ = 3.73 × 10^−04^) is involved in the conversion of angiotensin II into angiotensin III and the regulation of blood pressure^85^. Hence, in-development aminopeptidase inhibitors such as RB150 and angiotensin receptor blockers (ARBs) should be considered as potential therapies to investigate in MVP^86,87^.

### Discussion

**Fig. 7:**
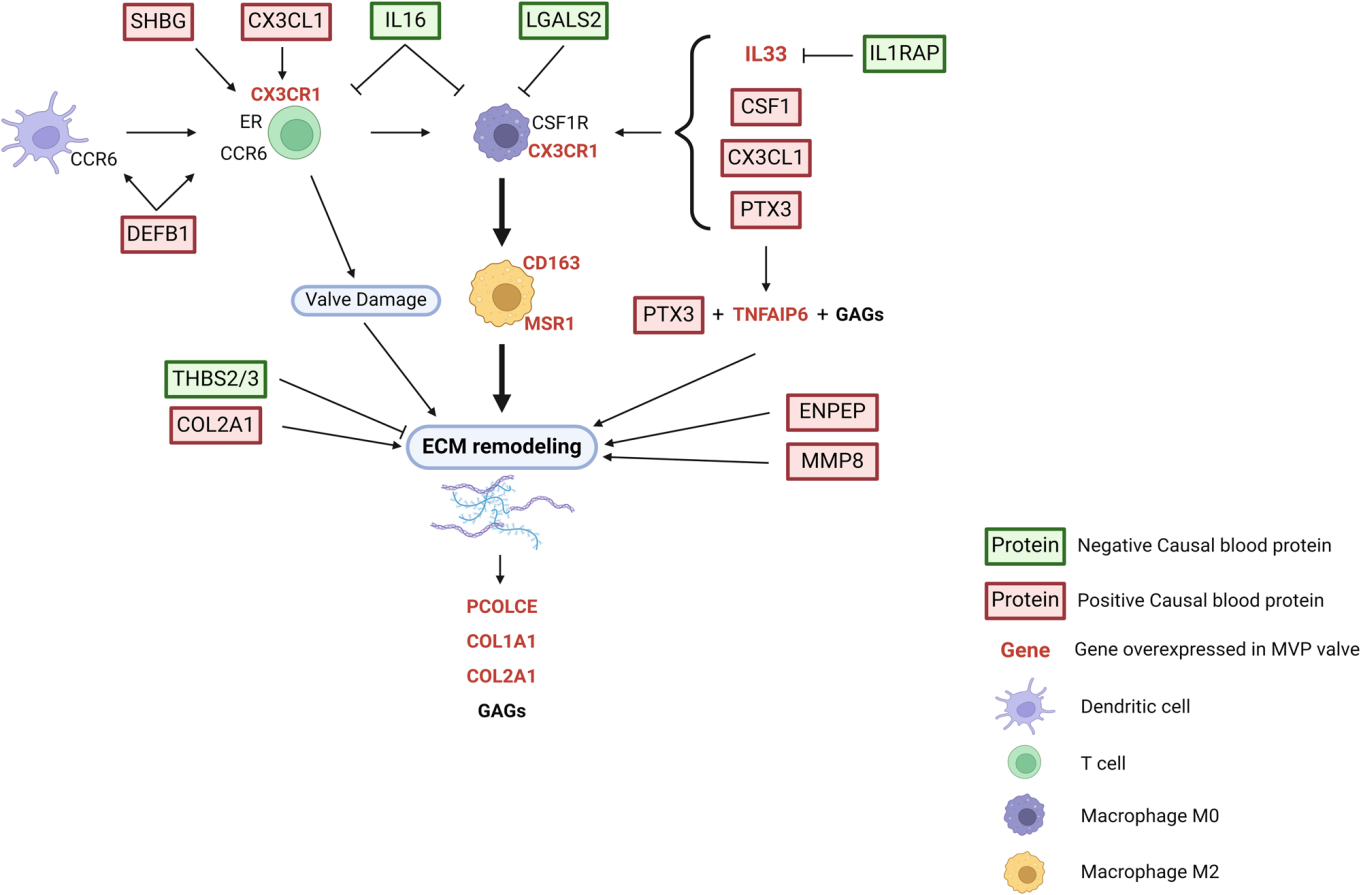
Integrative analysis of MVP results. Blood proteins in red and green rectangles are positively and negatively associated with MVP respectively. Genes written in red are overexpressed in MVP leaflets. Graph shows the implication of adaptative immune system through the activation of T cells, leading to valve damage and extra-cellular matrix (ECM) remodeling^110,111^. T cells are activated by different pathways. DEFB1 and its receptor CCR6 play a role in the activation of both dendritic cells and T cells^82^. SHBG increases the cytotoxic activity of T cells through their estrogen receptors (ER)^91^. T cells bearing the receptor of inflammatory cytokine CX3CL1 have an increased cytotoxic activity^90^. Graph also shows the importance of innate immune response via the polarization of M0 macrophages into M2 subtype, which are implicated in tissue repair and production of ECM^102^. CSF1, CX3CL1, PTX3 and IL33, which is overexpressed in MVP leaflets, are associated with the polarization of macrophages expressing CD163 and MSR1^64,98,103,110^. IL16, LGALS2 and ILRAP are negatively associated with the risk of MVP. IL16 dampen the immune response, notably by the inhibition of macrophage polarization^100^. LGALS2 hampers the polarization toward M2 macrophages in mice^104^. Soluble IL1RAP modulates the activity of its ligand IL33 by acting as a decoy factor^57^. ECM remodeling is impacted by PTX3, positively associated with MVP in MR, and TNFAIP6, overexpressed in MVP leaflets. PTX3 and TNFAIP6 interact with glycosaminoglycans (GAGs) (notably hyaluronan) in ECM^112,113^. COL2A1, which is positively associated with the risk of MVP, is a component of ECM. ENPEP and MMP8 could further impact the remodeling of ECM. THBS2 and THBS3 (THBS2/3) are negatively associated with MVP and are involved in the regulation of fibrillogenesis^114^. Figure generated with the help of Biorender.

By using an integrative cross-modality approach, we found that several molecules involved in both immunity and regulation of the ECM were associated with the development of MVP. Comprehensive assessment in MR identified that 33 blood proteins were robustly associated with the risk of MVP. Several predicted interactions between the blood proteins and MVLs may promote the M2 polarization of macrophages and the development of MVP. Figure 7 represents key mechanistic insights from the integrative analysis in MVP.

The immunome represents the collection of cell and molecular phenomes involved in the regulation of the immune response^88,89^. The present work using cross-modality data suggested that an immunome, characterized by blood proteins and dysregulated cell population in MVLs, participate to the pathogenesis of MVP. Among the MVP-associated blood proteins, a small group have a documented activity on the adaptative immune response through the regulation of T cells. DEFB1 is involved in the recruitment of dendritic cells and T cells^82^. CX3CL1 and its overexpressed receptor in MVP CX3CR1 are linked to increased cytotoxic activity of T cells^90^. SHBG, a sex hormone transporter can promote cytotoxic activity of T cells through their estrogen receptors and cause damage to heart valves^91,92^. Finally, IL16, an interleukin negatively associated with the risk of MVP in MR, could impact on several immune processes including the function of lymphocytes. To this effect, studies have underlined that IL16 promotes the anergy of T cells and dampen the immune response^93,94^. These results are consistent with the DCQ analysis conducted on control and MVP MVLs, which showed a higher proportion of dendritic and T cells in pathological condition. Collectively, these data have underlined the potential role of the adaptative immune response in MVP.

Previous studies showed that heart valves are populated by several immune cell types including resident macrophages as well as dendritic cells^14,15^ and observational histopathological studies conducted on surgically explanted human myxomatous mitral valves have showed a higher proportion of macrophages expressing mannose receptors^13,14^. A fine-grained analysis of our data has highlighted the rise in the proportion of M2 macrophages in MVP as well as the increased activity of cytokines known for promoting the maintenance and activation of macrophages in MVLs. Among these proteins, CSF1 (M-CSF) is a hematopoietic colony-stimulating factor involved in the development of yolk sac macrophages, the renewal of tissue resident macrophages as well as the polarization toward a M2-like phenotype^69,95–97^. Blood CSF1 was positively associated to MVP in MR analysis. The co-activity of TGF-beta family members and CSF1 in MVP suggests that the cytokine activity profile in MVLs may drive a M2 polarization with tissue remodeling properties^78^ (M2c). Other blood proteins identified in MR analysis supports the potential role of M2 macrophages in MVP. CX3CL1, a blood protein positively associated with MVP, promotes the polarization of macrophages into a M2 phenotype^98^. Of note, the expression of its receptor *CX3CR1*, a marker of cardiac resident macrophage^99^, was overexpressed in MVLs from MVP subjects. IL16, which was negatively associated with MVP development, is a known negative regulator of M2 polarization^100^. Furthermore, MVP overexpressed gene *IL33* is a target of the decoy receptor sIL1RAP, which was negatively associated with the risk of MVP. IL33 is produced by M2 macrophages and promotes the polarization of these cells in a feed forward mechanism^64^. M2c macrophages also express TGF-β and are involved in the remodeling and degradation of the ECM^101,102^. PTX3, a protein positively associated to the risk of MVP, promotes the expression of TGF-β in THP-1^103^, a human macrophage cell line. LGALS2, a protein negatively associated with the risk of MVP has been shown to impede the polarization toward M2 macrophages in mice^104^.

Collectively these data strongly militate for a role of reparative M2 macrophages in the pathogenesis of MVP. These hypotheses are supported by experiments conducted in rodent model. In *Fbn1* deficient mice, which recapitulate several features of the Marfan syndrome including a myxomatous valve degeneration, the ablation of circulating monocyte resulted in less valve macrophages and reduced the thickening of the MVLs^105^.

Cross-modality data integration identified some actionable targets to consider for investigation in follow-up studies. We found that circulating ENPEP (glutamyl aminopeptidase), which promotes the conversion of angiotensin II into angiotensin III, was associated with MVP. Hence, compounds under investigation inhibiting aminopeptidase or drug repurposing of ARBs should be considered in MVP^106^. In isolated human mitral valve interstitial cells, the blockade of angiotensin receptors reduced TGF-β-induced expression of activation markers^12^. Also, immunomodulatory interventions should be explored as we identified several molecules involved in immune regulation. Under development CCR6 blockers could be considered in functional follow-up studies in MVP as DEFB1 is an agonist of CCR6^81^. Pexidartinib, which inhibits CSF1R, is approved for the treatment of tenosynovial giant cell tumor^79^ and could be considered in MVP. Inhibition of molecules such as IL33 and CX3CR1, which are overexpressed in MVP, and promote M2 polarization, should be considered as actionable targets. To this effect, MR identified that circulating CX3CL1 and sIL1RAP, which interact with CX3CR1 and IL33, were positively and negatively associated with the risk of MVP, respectively.

The present study has some limitations. Causal inference using MR is a robust method, but may be prone to horizontal pleiotropy, which occurs when an instrument (gene variant) is associated to the outcome by using an alternative pathway^107^. However, we minimized the risk of pleiotropy by implementing several filters and sensitivity analyses. The Cochran’s Q and Egger intercept tests, which were implemented in this work, are robust methods to identify heterogeneity and pleiotropy in MR^108,109^. Also, the implementation of weighted median MR, which is resistant to invalid instruments, provided further robustness to the present findings^36^. As we used GWAS data representing large-scale population, the present inference is likely limited to non-syndromic form of MVP. Also, it was not possible to examine association with MVP subtypes (i.e., Barlow disease and fibroelastic deficiency).

Enrichment of receptors based on the Human Protein Atlas^40^ has inherent limitations as the dataset for the heart is limited to the myocardium and does not include heart valve specific gene expression. Also, we cannot exclude the possibility that some of the candidate ligands may act through receptors expressed in non-cardiac tissues. Some of the bioinformatic analyses such as DCQ and cytokine activity profiling rely on the assumption that source data is reliable for statistical inference in specific conditions (e.g., heart valve disorders). As such, some findings from this work are hypothesis-generating and warrant functional follow-up studies. To this effect, inference of ligand-receptor interactions and cytokine activity will need further functional assessment in order to provide detailed mechanistic insights.

Herein, analyses relying on the integration of large-scale cross-modality data provide the first evidence for a causal role of the circulating proteome and immunome in the development of MVP. Proteome-wide MR analyses pinpoint key molecules in circulation that impact the risk of MVP. These data provide a roadmap for downstream functional and clinical investigations.

### Supplementary information

Supplementary information

Description of Additional Supplementary Files

Supplementary Data 1

Supplementary Data 2

Supplementary Data 3

Supplementary Data 4

Supplementary Data 5

Supplementary Data 6

Supplementary Data 7

Supplementary Data 8

Supplementary Data 9

Supplementary Data 10

Supplementary Data 11

Supplementary Data 12

Supplementary Data 13

Supplementary Data 14

Supplementary Data 15

Supplementary Data 16

Supplementary Data 17

Supplementary Data 18

Supplementary Data 19

Reporting Summary

## Data Availability

All data used in this study are publicly available for download. Summary statistics for GWAS MVP^11^ are available on the cardiovascular disease knowledge portal (https://cvd.hugeamp.org/). Summary statistics for deCODE^29^ and SCALLOP^28^ studies are available on their respective study websites (https://www.decode.com/summarydata/ and https://zenodo.org/record/2615265#.YVHylrhPGBY). Microarray dataset is available on Gene Expression Omnibus, accession code GSE109744^60^. LM22^68^ dataset is publicly available (https://cibersortx.stanford.edu/). InnateDB^39^ dataset can be accessed from the InnateDB portal (https://www.innatedb.com/redirect.do?go=downloadCurated). Data in the Human Protein Atlas from the Tissue cell type section and the Heart muscle are available online on the Human Protein Atlas (HPA)^40^ website (https://www.proteinatlas.org/humanproteome/tissue+cell+type). Source data for all figures are available as supplementary data (Fig. 2: Supplementary Data 8 and 9; Fig. 3: Supplementary Data 10 and 11; Fig. 4: Supplementary Data 12, 13 and 14; Fig. 5: Supplementary Data 15, 16, 17, 18 and 19. All other data are available from the corresponding author on reasonable request.
