# Supplementary material for: Mendelian randomization reveals interactions of the blood proteome and immunome in mitral valve prolapse
Source: Commun Med (Lond). 2024 Jun 6;4:108. doi: 10.1038/s43856-024-00530-x (PMC11156961; doi:10.1038/s43856-024-00530-x)
Supplement: Supplementary file 1 — Supplementary information [file 43856_2024_530_MOESM1_ESM.pdf]

Supplementary information

Table of Contents

Supplementary Table 1. Summary of Steiger filtering for candidate blood proteins (deCODE)

Supplementary Table 2. Summary of Steiger filtering for candidate blood proteins (SCALLOP)

Supplementary Table 3. Vote rank pathway enrichment in Kyoto Encyclopedia of Gene and Genome

Supplementary Table 4. Drug Gene interaction Database (DGIdb) for the MVP causal proteins

Supplementary Table 1. Summary of Steiger filtering for candidate blood proteins (deCODE)

Steiger filtering					
**Aptamer**	**Gene symbol**	**Uniprot id**	**Direction**	**P-value Steiger**	**Filtered SNPs**
ACAT2.17341.89	ACAT2	Q9BWD1	TRUE	<10E-300	NA
ALAD.15523.9	ALAD	P13716	TRUE	<10E-300	NA
ALDH2.18381.16	ALDH2	P05091	TRUE	1,45E-24	NA
APOA5.15363.32	APOA5	Q6Q788	TRUE	<10E-300	NA
APOL3.16823.75	APOL3	O95236	TRUE	<10E-300	NA
CNP.6609.22	CNP	P09543	TRUE	5,73E-29	NA
COL2A1.18875.125	COL2A1	P02458	TRUE	<10E-300	NA
CRISPLD2.5691.2	CRISPLD2	Q9H0B8	TRUE	<10E-300	NA
DEFB1.6629.3	DEFB1	P60022	TRUE	<10E-300	NA
ENPEP.15558.63	ENPEP	Q07075	TRUE	<10E-300	NA
FER.4220.39	FER	P16591	TRUE	7,54E-30	NA
GM2A.15441.6	GM2A	P17900	TRUE	<10E-300	NA
HP.3054.3	HP	P00738	TRUE	<10E-300	NA
IGFBP3.2571.12	IGFBP3	P17936	TRUE	<10E-300	NA
IL1RAP.14048.7	IL1RAP	Q9NPH3	TRUE	<10E-300	NA
LGALS2.3033.57	LGALS2	P05162	TRUE	<10E-300	NA
LPO.4801.13	LPO	P22079	TRUE	<10E-300	NA
MAGI2.14066.49	MAGI2	Q86UL8	TRUE	<10E-300	NA
MASP1.3605.77	MASP1	P48740	TRUE	8,94E-59	NA
MFAP2.9294.45	MFAP2	P55001	TRUE	<10E-300	NA
MMP8.9172.69	MMP8	P22894	TRUE	<10E-300	NA
MTHFSD.19129.15	MTHFSD	Q2M296	TRUE	<10E-300	NA
PLXNA1.9005.16	PLXNA1	Q9UIW2	TRUE	<10E-300	NA
SHBG.4929.55	SHBG	P04278	TRUE	<10E-300	NA
STAT6.10372.18	STAT6	P42226	TRUE	2,39E-30	NA
TAPBPL.6364.7	TAPBPL	Q9BX59	TRUE	<10E-300	NA
THBS2.14111.15	THBS2	P35442	TRUE	<10E-300	NA
THBS3.8982.65	THBS3	P49746	TRUE	2,17E-78	NA

Supplementary Table 2. Summary of Steiger filtering for candidate blood proteins (SCALLOP)

Steiger filtering				
**Gene Symbol**	**UniProt id**	**Direction**	**P-value Steiger**	**Filtered SNPs**
CSF1	P09603	TRUE	3,41E-131	NA
CTSD	P07339	TRUE	<10E-300	NA
CX3CL1	P78423	TRUE	2,31E-223	NA
IL16	Q14005	TRUE	2,99E-192	NA
PTX3	P26022	TRUE	7,33E-26	NA

Supplementary Table 3. Vote rank pathway enrichment in Kyoto Encyclopedia of Gene and Genome

Term	Overlap	P-value	Adjusted P-value	Odds Ratio	Genes
Cytokine-cytokine receptor interaction	4/295	5,37E-04	0,024736386	12,29803187	CSF1;IL16;IL1RAP;CX3CL1
Tryptophan metabolism	2/42	0,001355132	0,024736386	41,52916667	ALDH2;ACAT2
Fatty acid degradation	2/43	0,001420102	0,024736386	40,51422764	ALDH2;ACAT2
Pyruvate metabolism	2/47	0,001694616	0,024736386	36,90555556	ALDH2;ACAT2
Valine, leucine and isoleucine degradation	2/48	0,001766885	0,024736386	36,10144928	ALDH2;ACAT2
Lysine degradation	2/63	0,003022777	0,035265727	27,20355191	ALDH2;ACAT2
PPAR signaling pathway	2/74	0,004144016	0,041440163	23,03472222	UBC;APOA5
Viral protein interaction with cytokine and cytokine receptor	2/100	0,007438995	0,064770153	16,90136054	CSF1;CX3CL1
Th17 cell differentiation	2/107	0,008475229	0,064770153	15,76904762	STAT6;IL1RAP
TNF signaling pathway	2/112	0,009252879	0,064770153	15,04848485	CSF1;CX3CL1
PI3K-Akt signaling pathway	3/354	0,010572255	0,067277986	7,292084727	COL2A1;CSF1;MAGI2
Synthesis and degradation of ketone bodies	1/10	0,012926986	0,075407416	88,73333333	ACAT2
Pantothenate and CoA biosynthesis	1/21	0,026961136	0,12766912	39,908	ALDH2
Histidine metabolism	1/22	0,028227412	0,12766912	38,00571429	ALDH2
Terpenoid backbone biosynthesis	1/22	0,028227412	0,12766912	38,00571429	ACAT2
Rap1 signaling pathway	2/210	0,030226609	0,12766912	7,919070513	CSF1;MAGI2
Maturity onset diabetes of the young	1/26	0,033276689	0,12766912	31,9184	HNF1A
Butanoate metabolism	1/28	0,035791859	0,12766912	29,55111111	ACAT2
Ascorbate and aldarate metabolism	1/30	0,038300736	0,12766912	27,51034483	ALDH2
beta-Alanine metabolism	1/30	0,038300736	0,12766912	27,51034483	ALDH2
Glyoxylate and dicarboxylate metabolism	1/30	0,038300736	0,12766912	27,51034483	ACAT2
Propanoate metabolism	1/34	0,043299673	0,137771687	24,17090909	ACAT2
Fat digestion and absorption	1/43	0,054456098	0,155483069	18,98285714	ACAT2
Porphyrin and chlorophyll metabolism	1/43	0,054456098	0,155483069	18,98285714	ALAD
MAPK signaling pathway	2/294	0,055529667	0,155483069	5,617009132	CSF1;IL1RAP
Arginine and proline metabolism	1/50	0,063046725	0,169741183	16,26530612	ALDH2
Glycerolipid metabolism	1/61	0,076394849	0,197851976	13,276	ALDH2

Inflammatory bowel disease	1/65	0,081203226	0,197851976	12,44375	STAT6
Glycolysis / Gluconeogenesis	1/67	0,08359838	0,197851976	12,06545455	ALDH2
Mitophagy	1/68	0,084793704	0,197851976	11,88477612	UBC
Adherens junction	1/71	0,088370688	0,198510972	11,37371429	FER
p53 signaling pathway	1/73	0,090747873	0,198510972	11,05666667	IGFBP3
Complement and coagulation cascades	1/85	0,1048863	0,210070426	9,471428571	MASP1
ECM-receptor interaction	1/88	0,108387738	0,210070426	9,143448276	COL2A1
Th1 and Th2 cell differentiation	1/92	0,113035841	0,210070426	8,73978022	STAT6
Rheumatoid arthritis	1/93	0,114194221	0,210070426	8,644347826	CSF1
IL-17 signaling pathway	1/94	0,115351147	0,210070426	8,550967742	ELAVL1
Staphylococcus aureus infection	1/95	0,116506619	0,210070426	8,459574468	MASP1
Inflammatory mediator regulation of TRP channels	1/98	0,119964336	0,210070426	8,196701031	IL1RAP
Hematopoietic cell lineage	1/99	0,121114014	0,210070426	8,112653061	CSF1
Protein digestion and absorption	1/103	0,125698301	0,210070426	7,792941176	COL2A1
Pathways of neurodegeneration	2/475	0,126042256	0,210070426	3,435694151	CSF1;UBC
Sphingolipid signaling pathway	1/119	0,143806588	0,225440912	6,730847458	CTSD
Growth hormone synthesis, secretion and action	1/119	0,143806588	0,225440912	6,730847458	IGFBP3
AMPK signaling pathway	1/120	0,1449263	0,225440912	6,67394958	ELAVL1
Osteoclast differentiation	1/127	0,152724954	0,229113647	6,300952381	CSF1
Lysosome	1/128	0,153833449	0,229113647	6,251023622	CTSD
Autophagy	1/137	0,16374735	0,23224451	5,834705882	CTSD
Estrogen signaling pathway	1/137	0,16374735	0,23224451	5,834705882	CTSD
Ubiquitin mediated proteolysis	1/140	0,167027094	0,23224451	5,707913669	UBC
Apoptosis	1/142	0,169206714	0,23224451	5,626382979	CTSD
Cellular senescence	1/156	0,184311231	0,242713477	5,114580645	IGFBP3
Necroptosis	1/159	0,187513383	0,242713477	5,016708861	STAT6
JAK-STAT signaling pathway	1/162	0,190703446	0,242713477	4,922484472	STAT6
Hepatitis B	1/162	0,190703446	0,242713477	4,922484472	STAT6
Tuberculosis	1/180	0,209592399	0,259939242	4,423463687	CTSD
Axon guidance	1/182	0,211664811	0,259939242	4,374143646	PLXNA1

Transcriptional misregulation in cancer	1/192	0,221948746	0,260131685	4,143036649	IGFBP3
Chemokine signaling pathway	1/192	0,221948746	0,260131685	4,143036649	CX3CL1
Kaposi sarcoma-associated herpesvirus infection	1/193	0,222970015	0,260131685	4,12125	UBC
Focal adhesion	1/201	0,231093897	0,263191047	3,9548	COL2A1
Diabetic cardiomyopathy	1/203	0,23311207	0,263191047	3,915247525	CTSD
Human cytomegalovirus infection	1/225	0,254978479	0,28194853	3,526785714	CX3CL1
Coronavirus disease	1/232	0,26180935	0,28194853	3,418701299	MASP1
Ras signaling pathway	1/232	0,26180935	0,28194853	3,418701299	CSF1
Shigellosis	1/246	0,275290874	0,290603237	3,221061224	UBC
Parkinson disease	1/249	0,278148812	0,290603237	3,181612903	UBC
Human papillomavirus infection	1/331	0,352200057	0,362558882	2,381090909	COL2A1
Alzheimer disease	1/369	0,383985761	0,389550772	2,131086957	CSF1
Pathways in cancer	1/531	0,503450174	0,503450174	1,467471698	STAT6

Supplementary Table 4. Drug Gene interaction Database (DGIdb) for the MVP causal proteins

Gene Symbol	drug	Interaction types	Sources	pmids
CX3CL1	ATEZOLIZUMAB	###	CIViC	25428504
CSF1	PEXIDARTINIB	###	CIViC	26222558
CSF1	EMACTUZUMAB	###	TTD	###
APOA5	LOVASTATIN	###	PharmGKB	19530961
APOA5	FENOFIBRATE	###	PharmGKB	17431185|19057464|19056598
APOA5	SIMVASTATIN	###	PharmGKB	19530961
APOA5	ATORVASTATIN	###	PharmGKB	19530961
COL2A1	OCRIPLASMIN	###	ChemblInteractions	###
COL2A1	COLLAGENASE CLOSTRIDIUM HISTOLYTICUM	###	ChemblInteractions|TEND	###
FER	HESPERADIN	inhibitor	DTC	19035792
FER	ALISERTIB	###	DTC	###
FER	SOTRASTAURIN	###	DTC	###
FER	CENISERTIB	###	DTC	###
FER	LINIFANIB	###	DTC	###
FER	GW441756X	###	DTC	###
FER	CEDIRANIB	###	DTC	###
FER	R-406	###	DTC	###
FER	RG-1530	###	DTC	###
FER	CYC-116	###	DTC	###
FER	ILORASERTIB	###	DTC	###
FER	SP-600125	###	DTC	###
FER	ENTRECTINIB	###	DTC	###
FER	OSI-632	###	DTC	###
IGFBP3	CELECOXIB	###	PharmGKB	22336956
IGFBP3	FLUOROURACIL	###	PharmGKB	20860465
LPO	DIETHYLSTILBESTROL	###	NCI	10994878
LPO	T-817 MALEATE	###	TTD	###

LPO	RAXOFELAST	###	TTD	###
MMP8	DOXYCYCLINE	inhibitor	ChemblInteractions	###
MMP8	DOXYCYCLINE HYCLATE	inhibitor	ChemblInteractions	###
MMP8	DOXYCYCLINE CALCIUM	inhibitor	ChemblInteractions	###
MMP8	COLLAGENASE CLOSTRIDIUM HISTOLYTICUM	###	TdgClinicalTrial	###
MMP8	CIPEMASTAT	###	TTD	###
SHBG	NORGESTREL	###	NCI	573191
SHBG	DROLOXIFENE	###	NCI	7561649
SHBG	CHEMBL464631	###	DTC	11575952
SHBG	LISINOPRIL	###	NCI	11872207
SHBG	INSULIN	###	NCI	7593430
STAT6	CHEMBL1374370	###	DTC	###
STAT6	CHEMBL516616	###	DTC	###
STAT6	CHEMBL1481974	###	DTC	###
STAT6	CHEMBL429095	###	DTC	###
STAT6	CHEMBL605003	###	DTC	###
STAT6	CHEMBL72365	###	DTC	###
STAT6	PURVALANOLA	###	DTC	###
STAT6	CHEMBL363332	###	DTC	###
STAT6	SANGUINARIUM	###	DTC	###
STAT6	INDOMETHACIN	###	NCI	18832692
STAT6	ELLIPTECINE	###	DTC	###
STAT6	DOXORUBICIN HYDROCHLORIDE	###	DTC	###
