# Supplementary material for: Mendelian randomization reveals interactions of the blood proteome and immunome in mitral valve prolapse
Source: Commun Med (Lond). 2024 Jun 6;4:108. doi: 10.1038/s43856-024-00530-x (PMC11156961; doi:10.1038/s43856-024-00530-x)
Supplement: Supplementary file 2 — Description of Additional Supplementary Files [file 43856_2024_530_MOESM2_ESM.docx]

**Description of Additional Supplementary Files**

File name- Supplementary Data 1.

File description- Mendelian randomization of blood proteins on the risk of MVP, MAF ≥ 0.01 (deCODE). Results of MR analysis for the candidate blood proteins associated with the risk of MVP in deCODE cohort. Table includes: Aptamers id; protein gene symbol; the number of SNPs used as IVs in MR for each protein; the median, minimum and maximum F-statistic values for IVs; the effect size beta, standard error and P-values for inverse variance weighted; FDR and Bonferroni multiple correction for inverse variance weighted method; heterogeneity test (Cochran’s Q test); pleiotropy test (Egger P-value intercept).

File name- Supplementary Data 2.

File description- Mendelian randomization of blood proteins on the risk of MVP, Weighted Median sensitivity test, MAF ≥ 0.01 (deCODE). Results of MR analysis for the candidate blood proteins associated with the risk of MVP in deCODE cohort. Table includes: Aptamers id; protein gene symbol; corresponding UniProt id; the number of SNPs used as IVs in MR for each protein; the median, minimum and maximum F-statistic values for IVs; the effect size beta, standard error and Pvalues for inverse variance weighted and weighted median methods; FDR multiple correction for inverse variance weighted and weighted median methods; Bonferroni multiple correction for inverse variance weighted ; heterogeneity test (Cochran’s Q test); pleiotropy test (Egger P-value intercept).

File name- Supplementary Data 3.

File description- Mendelian randomization of blood proteins on the risk of MVP, MAF ≥ 0.01 (SCALLOP). Results of MR analysis for the candidate blood proteins associated with the risk of MVP in SCALLOP cohort. Table includes: protein gene symbol; the number of SNPs used as IVs in MR for each protein; the median, minimum and maximum F- statistic values for IVs; the effect size beta, standard error and P-values for inverse variance weighted; FDR and Bonferroni multiple correction for inverse variance weighted method; heterogeneity test (Cochran’s Q test); pleiotropy test (Egger P-value intercept).

File name- Supplementary Data 4

File description- Mendelian randomization of blood proteins on the risk of MVP, Weighted Median sensitivity test, MAF ≥ 0.01 (SCALLOP). Results of MR analysis for the candidate blood proteins associated with the risk of MVP in SCALLOP cohort. Table includes: protein gene symbol; corresponding UniProt id; the number of SNPs used as IVs in MR for each protein; the median, minimum and maximum Fstatistic values for IVs; the effect size beta, standard error and P-values for inverse variance weighted and weighted median methods; FDR multiple correction for inverse variance weighted and weighted median methods; Bonferroni multiple correction for inverse variance weighted ; heterogeneity test (Cochran’s Q test); pleiotropy test (Egger Pvalue intercept).

File name- Supplementary Data 5.

File description- Overview of common proteins in deCODE and SCALLOP MR studies. Table summarizing nominally significant proteins common in both MR analyses using deCODE and SCALLOP as exposure. Table includes: protein gene symbol; corresponding UniProt id; the effect size beta, standard error and P-values for inverse variance weighted; FDR multiple correction for inverse variance weighted method.

File name- Supplementary Data 6.

File description- Mendelian randomization of blood proteins on the risk of MVP, MAF ≥ 0.05 (deCODE). Results of MR analysis for the candidate blood proteins associated with the risk of MVP in deCODE cohort. Table includes: Aptamers id; protein gene symbol; corresponding UniProt id; the number of SNPs used as IVs in MR for each protein; the median, minimum and maximum Fstatistic values for IVs; the effect size beta, standard error and P-values for inverse variance weighted method; FDR multiple correction for inverse variance weighted method; heterogeneity test (Cochran’s Q test); pleiotropy test (Egger P-value intercept).

File name- Supplementary Data 7.

File description- Mendelian randomization of blood proteins on the risk of MVP, MAF ≥ 0.05 (SCALLOP). Results of MR analysis for the candidate blood proteins associated with the risk of MVP in SCALLOP cohort. Table includes: protein gene symbol; corresponding UniProt id; the number of SNPs used as IVs in MR for each protein; the median, minimum and maximum F-statistic values for IVs; the effect size beta, standard error and Pvalues for inverse variance weighted method; FDR multiple correction for inverse variance weighted method; heterogeneity test (Cochran’s Q test); pleiotropy test (Egger P-value intercept).

File name- Supplementary Data 8.

File description- Input Table Forest plot deCODE (Figure 2a). Table summarizing MR results for the 28 causal candidates identified using deCODE as exposure. Table was used for Figure 2a, and includes: Aptamers id; protein gene symbol; Uniprot id; the number of SNPs used as IVs in MR for each protein; the median, minimum and maximum Fstatistic values for IVs; the effect size beta, standard error and P-values for inverse variance weighted; corresponding Odd ratio, lower limits and upper limits; FDR and Bonferroni multiple correction for inverse variance weighted method; heterogeneity test (Cochran’s Q test); pleiotropy test (Egger P-value intercept).

File name- Supplementary Data 9.

File description- Input Table Forest plot SCALLOP (Figure 2b). Table summarizing MR results for the 5 causal candidates identified using SCALLOP as exposure. Table was used for Figure 2b, and includes: protein gene symbol; Uniprot id; the number of SNPs used as IVs in MR for each protein; the median, minimum and maximum Fstatistic values for IVs; the effect size beta, standard error and P-values for inverse variance weighted; corresponding Odd ratio, lower limits and upper limits; FDR and Bonferroni multiple correction for inverse variance weighted method; heterogeneity test (Cochran’s Q test); pleiotropy test (Egger P-value intercept).

File name- Supplementary Data 10.

File description- Input Table Protein interaction network (Figure 3a). Table of protein-protein interactions extracted from InnateDB using the causal proteins as seeds. Table was used for Figure 3a and shows: emitting node (node_1), type of linkage (link_type) and receiving node (node_2). Abbreviation pp correspond to protein-protein.

File name- Supplementary Data 11.

File description- Input Table Enrichment GO Biological Process (Figure 3b). Results of gene ontology enrichment for all nodes in MVP protein/protein interaction network by using the biological process dataset (GO biological process). Table was used for Figure 3b and shows enrichment term and associated overlap, P-value, adjusted P-value, odds ratio and enriched gene list.

File name- Supplementary Data 12.

File description- Input Table LigandReceptor pairs (bold are causal proteins) (Figure 4a). Table summarizing identified ligand-receptor pairs used in Figure 4a. Protein names in bold are causal candidates identified in MR analyses.

File name- Supplementary Data 13

File description- Input Table HPA heart cell enrichment (Figure 4b). Table summarizing enrichment of receptors for their respective cognate candidate blood ligands in 9 different cardiac cell-types from the heart dataset in the Human Protein Atlas. Table was used for Figure 4b and shows cell type, fold change (FC), P-value and associated -log P-value.

File name- Supplementary Data 14

File description- . Input Table differentially expressed genes (Control MVLs vs MVP MVLs) (Figure 4c, d). Table summarizing differentially expressed genes in mitral valve microarray dataset. Table was used in Figure 4c and d. Table shows Entrez id with corresponding gene symbols and full name, the log fold change MVP vs control (Log FC MVP vs CTL), the average expression, the Pvalue and corresponding adjusted P-value.

File name- Supplementary Data 15.

File description- Input Table principal component analysis for deconvoluted immune cells (Figure 5a). Table summarizing the digital cell quantification for 22 immune cell types in the microarray dataset. Samples 1 to 12 are control samples, samples 13 to 24 are MVP samples. Table was used for Figure 5a.

File name- Supplementary Data 16.

File description- Input Table Immune cell type proportion enrichment (Figure 5b). Table summarizing proportion enrichment for immune cell types in mitral valve leaflets control and MVP. Table was used in Figure 5b and shows mean cell proportion and standard deviation (SD) for control (CTL) and MVP conditions, P-values for proportion difference with corresponding -log P-values and false discovery rate (FDR).

File name- Supplementary Data 17.

File description- Correlation Matrix for cytokines activity (Figure 5c). Correlation matrix of cytokines activity in MVLs. Matrix was used to create dendrogram of similarity profiles of cytokine activity in MVLs (Figure 5c).

File name- Supplementary Data 18

File description-. Input Table ridgeline plot of subset-cytokine activity (Control MVLs vs MVP MVLs) (Figure 5d). Table summarizing the activity of 6 cytokines in control and MVP samples. Table was used to generate ridge line plot in Figure 5d and shows cytokine symbol, status of sample and activity score.

File name- Supplementary Data 19.

File description- Input Table Boxplot MSR1, CD163 (Figure 5e). Tables showing summarized results of differential expression of MSR1 and CD163 in response to treatment with CSF1. Table was used in generation of boxplots Figure 5e and shows individual experiments and adjusted log fold change (log(FC)) in each experiment.
