# Supplementary material for: Mendelian randomization reveals interactions of the blood proteome and immunome in mitral valve prolapse
Source: Commun Med (Lond). 2024 Jun 6;4:108. doi: 10.1038/s43856-024-00530-x (PMC11156961; doi:10.1038/s43856-024-00530-x)
Supplement: Supplementary file 22 — Reporting Summary [file 43856_2024_530_MOESM22_ESM.pdf]

nature portfolio

Corresponding author(s): Mathieu Patrick

Last updated by author(s): May 13, 2024

Reporting Summary

Nature Portfolio wishes to improve the reproducibility of the work that we publish. This form provides structure for consistency and transparency in reporting. For further information on Nature Portfolio policies, see our Editorial Policies and the Editorial Policy Checklist.

Statistics

For all statistical analyses, confirm that the following items are present in the figure legend, table legend, main text, or Methods section.

n/a	Confirmed	
☐	☒	The exact sample size (*n*) for each experimental group/condition, given as a discrete number and unit of measurement
☐	☒	A statement on whether measurements were taken from distinct samples or whether the same sample was measured repeatedly
☐	☒	The statistical test(s) used AND whether they are one- or two-sided<br>*Only common tests should be described solely by name; describe more complex techniques in the Methods section.*
☐	☒	A description of all covariates tested
☐	☒	A description of any assumptions or corrections, such as tests of normality and adjustment for multiple comparisons
☐	☒	A full description of the statistical parameters including central tendency (e.g. means) or other basic estimates (e.g. regression coefficient) AND variation (e.g. standard deviation) or associated estimates of uncertainty (e.g. confidence intervals)
☐	☒	For null hypothesis testing, the test statistic (e.g. *F*, *t*, *r*) with confidence intervals, effect sizes, degrees of freedom and *P* value noted<br>*Give P values as exact values whenever suitable.*
☒	☐	For Bayesian analysis, information on the choice of priors and Markov chain Monte Carlo settings
☐	☒	For hierarchical and complex designs, identification of the appropriate level for tests and full reporting of outcomes
☐	☒	Estimates of effect sizes (e.g. Cohen's *d*, Pearson's *r*), indicating how they were calculated

Our web collection on statistics for biologists contains articles on many of the points above.

Software and code

Policy information about availability of computer code

Data collection	Summary statistics for GWAS, cell traits, blood proteins and microarray are publicly available. Data were downloaded on appropriate websites. URLs are provided in the manuscript within the Data availability section.
Data analysis	Data analysis conducted in this manuscript are described with appropriate tools in the Methods section of the manuscript.<br>MR analyses were performed by using the Mendelian Randomization package.<br>Genomic coordinates in GRCh38 were converted in GRCh37 by using LiftOver tool from UCSC.<br>Gene ontology and pathway analyses were performed with Enrichr.<br>Network was extracted from the InnateDB, a dataset reporting curated protein-protein interactions.<br>ligand-receptor pairs were identified using currated dataset from Shao et al. (reference available in manuscript)<br>Analysis of differencial expression was conducted using R package Limma in ExpressAnalyst.<br>GSEA Analysis was conducted by using online tool Webgestalt.<br>Principal component analysis was performed in R package pcaMethods.<br>Digital cell quantification was performed by using python package GEDIT.<br>cytokine activity profiling analysis was conducted using Cytokine Signaling (CytoSig).<br>Potential accionable targets were identified using DGIdb, a repository of drug-gene pairs

For manuscripts utilizing custom algorithms or software that are central to the research but not yet described in published literature, software must be made available to editors and reviewers. We strongly encourage code deposition in a community repository (e.g. GitHub). See the Nature Portfolio guidelines for submitting code & software for further information.

Data

Policy information about availability of data

All manuscripts must include a data availability statement. This statement should provide the following information, where applicable:

- Accession codes, unique identifiers, or web links for publicly available datasets
- A description of any restrictions on data availability
- For clinical datasets or third party data, please ensure that the statement adheres to our policy

All data are available in the manuscript including in supplementary tables and figures. Summary statistics for GWAS, blood proteins are publicly available. Accession codes are available in the Methods section of the manuscript. URLs are provided in the manuscript within the Data availability section.

Human research participants

Policy information about studies involving human research participants and Sex and Gender in Research.

Reporting on sex and gender	data used in this manuscript did not report on sex and/or gender
Population characteristics	GWAS and pQTLs data were generated on population of European and Icelandic ancestry
Recruitment	No recruitment of human research participant were conducted during this study
Ethics oversight	Public data were used during this study, no ethics oversight were warranted

Note that full information on the approval of the study protocol must also be provided in the manuscript.

Field-specific reporting

Please select the one below that is the best fit for your research. If you are not sure, read the appropriate sections before making your selection.

☒ Life sciences ☐ Behavioural & social sciences ☐ Ecological, evolutionary & environmental sciences

For a reference copy of the document with all sections, see nature.com/documents/nr-reporting-summary-flat.pdf

Life sciences study design

All studies must disclose on these points even when the disclosure is negative.

Sample size	Study is based on publicly available data, no sample size was determined, all data were used for analysis.
Data exclusions	No data were excluded from presented analyses.
Replication	Mendelian randomization analysis were replicated in two pQTLs cohorts, deCODE and SCALLOP
Randomization	No random allocation of patients or samples were necessary during this study.
Blinding	Study is based on publicly available data, blinding to group allocation was conducted.

Reporting for specific materials, systems and methods

We require information from authors about some types of materials, experimental systems and methods used in many studies. Here, indicate whether each material, system or method listed is relevant to your study. If you are not sure if a list item applies to your research, read the appropriate section before selecting a response.

Materials & experimental systems

n/a	Involved in the study	
☒	☐	Antibodies
☒	☐	Eukaryotic cell lines
☒	☐	Palaeontology and archaeology
☒	☐	Animals and other organisms
☒	☐	Clinical data
☒	☐	Dual use research of concern

Methods

n/a	Involved in the study	
☒	☐	ChIP-seq
☒	☐	Flow cytometry
☒	☐	MRI-based neuroimaging
